# Experimental Investigation of Water Jet-Guided Laser Micro-Hole Drilling of C_f_/SiC Composites

**DOI:** 10.3390/ma17091975

**Published:** 2024-04-24

**Authors:** Binying Bao, Guangyi Zhang, Zhongan Chen, Yang Chao, Chunhai Guo, Wenwu Zhang

**Affiliations:** 1 College of Mechanical Engineering, Zhejiang University of Technology, Hangzhou 310023, China; baobinying@nimte.ac.cn (B.B.); chenzhongan@nimte.ac.cn (Z.C.); 2 Ningbo Institute of Materials Technology and Engineering, Chinese Academy of Sciences, Ningbo 315201, China; chaoyang@nimte.ac.cn (Y.C.); guochunhai@nimte.ac.cn (C.G.)

**Keywords:** water jet-guided laser, C_f_/SiC composites, micro-hole drilling, influencing factor, microstructure analysis

## Abstract

In this paper, water jet-guided laser (WJGL) drilling of $C_{f}/\mathrm{SiC}$ composites was employed and the effects of the processing parameters on the depth and quality of the micro-holes were systematically investigated. Firstly, the depth measurement showed that the increase in processing time and power density led to a significant improvement in micro-hole drilling depth. However, the enhancement of the water jet speed resulted in a pronounced decrease in the depth due to the phenomenon of water splashing. In contrast, the scanning speed, path overlap ratio, pulse frequency, and helium pressure exhibited less effect on the micro-hole depth. Secondly, the microstructural analysis revealed that the increase in power density resulted in the deformation and fracture of the carbon fibers, while the augmentation in water jet speed reduced the thermal defects. Finally, based on the optimization of the processing parameters, a micro-hole of exceptional quality was achieved, with a depth-to-diameter ratio of 8.03 and a sidewall taper of 0.72°. This study can provide valuable guidance for WJGL micro-hole drilling of $C_{f}/\mathrm{SiC}$ composites.

## 1. Introduction

In the aerospace field, lightweight design is crucial for reducing the overall mass of the vehicle and plays a pivotal role in improving structural strength and safety. By adopting lightweight design, not only can the launch cost be lowered, but also the thrust-to-weight ratio of the engine is improved [1,2,3]. $C_{f}/\mathrm{SiC}$ ceramic composites possess substantially lower density than superalloys and are characterized by high-temperature resistance, high specific strength, and oxidation resistance. Additionally, by introducing continuous fibers, $C_{f}/\mathrm{SiC}$ composites address the brittleness of ceramic materials and may replace metals as a new generation of high-temperature structural materials [4,5,6]. Thus far, $C_{f}/\mathrm{SiC}$ composites have been applied to components such as combustion chambers, heat shields, wing leading edges, rocket nozzles, etc., in the context of ultra-high-speed vehicles [7,8,9,10].

Achieving precision machining of $C_{f}/\mathrm{SiC}$ composites is necessary to meet the requirements of assembly and application. However, the high hardness, anisotropy, and inhomogeneity of the fibers and distribution of pores of $C_{f}/\mathrm{SiC}$ composites present great challenges for their processing [11]. Traditional machining for $C_{f}/\mathrm{SiC}$ composites includes cutting, milling, drilling, etc., which has the advantages of a simple process, wide application, and high machining efficiency [12]. However, cutting forces in the direction of the perpendicular fiber layup can lead to delamination defects due to the low interlaminar bond strength of the material [13]. During drilling, the fibers are pulled out of the substrate by the axial force and generate burrs. The carbon fibers are removed mainly by fracture, resulting in a rough machined surface [14]. Moreover, the tool is subject to heavy wear. To minimize the defects present in conventional machining, various non-traditional machining methods have been developed. Rotary ultrasonic machining provides lower cutting forces and reduces tearing defects on the hole surface [15,16]. Abrasive water jet machining offers negligible thermal effects but is prone to cracking and delamination [17]. Laser processing is characterized by no mechanical stress and high energy density. However, one of the main disadvantages of laser ablation is the heat-affected zone [18]. To minimize the thermal damage, short-pulse lasers and ultrashort-pulse lasers were employed for $C_{f}/\mathrm{SiC}$ composite processing. However, the cutting surfaces of millisecond and nanosecond lasers are characterized by the presence of large amounts of debris and recast layers [19,20]. Picosecond and femtosecond lasers may reduce oxide generation but prolong processing time [21,22].

Water jet-guided laser (WJGL) processing technology combines a laser and water jet with minimal thermal effect, strong machining capability, and high adaptability [23,24,25]. In recent years, WJGL has been applied to composite materials processing by many researchers. Marimuthu et al. [26] drilled silicon carbide-reinforced aluminum matrix composites with WJGL and obtained holes without molten layers. Wu et al. [27] investigated the effects of laser power, feed speed, and water jet speed on the depth and width of carbon fiber-reinforced plastic (CFRP) cuts and analyzed the relationship between the direction of carbon fiber arrangement and cutting damage. Moreover, the parallel path layered scanning method was utilized to achieve the 10 mm thickness CFRP cutting. Cheng et al. [28] introduced a novel coaxial helical gas-assisted technique to improve WJGL processing capability. Eventually, a Si$C_{f}/\mathrm{SiC}$ composite microgroove with a maximum depth-to-width ratio of 13.6 and without recast layers, fiber pullout, and delamination was achieved. Hu et al. [29] studied the effect of laser power, scanning speed, and fill spacing on WJGL grooving of Si$C_{f}/\mathrm{SiC}$ composites. The experimental results showed that the processing parameters significantly affected the ablation depth, volume, and surface morphology. Therefore, different processing efficiency and quality requirements should be considered when selecting processing parameters.

Given the results of the literature review, the problem of high-quality deep-hole drilling of $C_{f}/\mathrm{SiC}$ composites requires an urgent solution, while the research on WJGL processing for $C_{f}/\mathrm{SiC}$ composites still lacks a detailed report. Therefore, this paper systematically investigates the effects of parameters such as the laser, water jet, and scanning path on the depth and morphology of WJGL micro-hole drilling of $C_{f}/\mathrm{SiC}$ composites. Based on optimizing the processing parameters, a high-quality micro-hole with a depth-to-diameter ratio of 8.03 and a depth of 4.1 mm was achieved. These micro-holes processed by WJGL can be applied in aerospace engines and brake disks. This paper presents a detailed analysis of the process of WJGL drilling of $C_{f}/\mathrm{SiC}$ composites and the mechanism of each factor, which can provide valuable guidance for high-quality deep-hole drilling.

## 2. Materials and Methods

### 2.1. Materials

**Figure 1:**
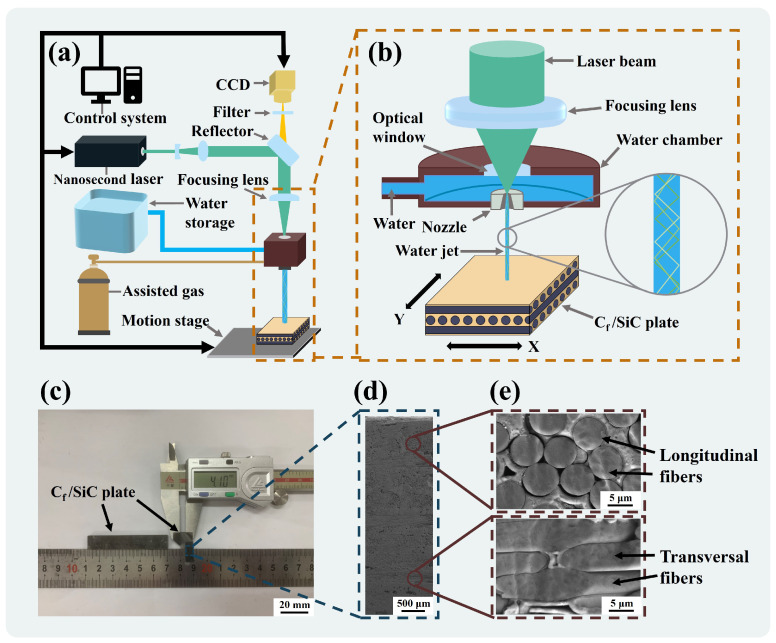
Water jet-guided laser (WJGL) processing system and experimental materials. (**a**) Schematic diagram of the WJGL processing system. (**b**) Schematic diagram of the principle of WJGL processing. (**c**) Macroscopic morphology of the $C_{f}/\mathrm{SiC}$ composites. (**d**) Cross-section morphology of the $C_{f}/\mathrm{SiC}$ composites. (**e**) Microstructure of the transversal and longitudinal carbon fibers.

The material used in the experiments was $C_{f}/\mathrm{SiC}$ ceramic composites with a 3-dimensional (3D) needle-punched structure (Zhejiang Hangyin New Material Technology Co., Hangzhou, China) measuring 58.2 mm × 10.0 mm × 4.1 mm, as shown in Figure 1c. The $C_{f}/\mathrm{SiC}$ composites consist of SiC matrix, carbon fibers, and pyrolytic carbon interface layer. Figure 1d shows its cross-sectional morphology, and the layered stacked carbon fibers can be divided into transversal and longitudinal carbon fibers, as shown in Figure 1e. The diameter of the carbon fiber is about 6–8 µm, and the volume fraction is about 40%. The characteristic parameters of $C_{f}/\mathrm{SiC}$ composites at room temperature are shown in Table 1.

### 2.2. WJGL System and Processing Principle

The experimental platform was a self-developed WJGL processing system. As shown in Figure 1a, the processing system mainly consists of the following components: control system, nanosecond laser, optical path system (including reflection lens, beam-expanding lens, focusing lens, and dichroic lens), charge coupled device (CCD), high-pressure water supply system, auxiliary gases, and motion stage. The laser source was a solid-state nanosecond laser with a wavelength of 532 nm. The auxiliary gas was helium, which coaxially surrounded the water jet to reduce the friction between the surface of the water jet and the air.

Figure 1b shows the principle of WJGL processing. First, the water supply system provides high-pressure deionized water into the water chamber, which is injected at the nozzle and forms a steady micro-water-jet. Then, the laser is coaxially aligned with the water jet and is focused through a focusing lens into the nozzle to couple with the water jet. The coupling error is reduced by CCD observation. Finally, the laser continuously undergoes total reflection in the water jet and is transmitted to the material to achieve processing.

### 2.3. Experimental Design

The effects of scanning speed, path overlap ratio, pulse frequency, helium pressure, processing time, power density, and water jet speed on the micro-hole depth and morphology of WJGL drilling of $C_{f}/\mathrm{SiC}$ composites were experimentally investigated. The experimental parameters are shown in Table 2. The laser power density *I* is calculated by the following equation:

(1) $$I=\frac{P_{\mathrm{avg}}}{f\tau A}$$

where $P_{avg}$ is the average laser power, *f* is the pulse frequency, *A* is the irradiated area of the WJGL, and τ is the pulse width.

In pulsed lasers, power density represents the amount of energy irradiated per unit time by a single laser pulse per unit area of the target material. Power density typically determines whether the material reaches a threshold for destruction, ablation, and other effects.

During the experiments, the changes in the WJGL irradiated area and pulse width were negligible. Thus, the power density was proportional to the average laser power and inversely proportional to the pulse frequency. In the single-factor experiments, the other parameters were held constant, where the power density was 0.10 GW/cm², the pulse frequency was 10 kHz, the water jet speed was 100 m/s, the scanning speed was 0.3 mm/s, the path overlap ratio was 50%, the helium pressure was 5 kPa, and the processing time was 45 s. The holes obtained in the experiments were blind. Each set of experiments was conducted three times.

### 2.4. Drilling Strategy and Characterization

**Figure 2:**
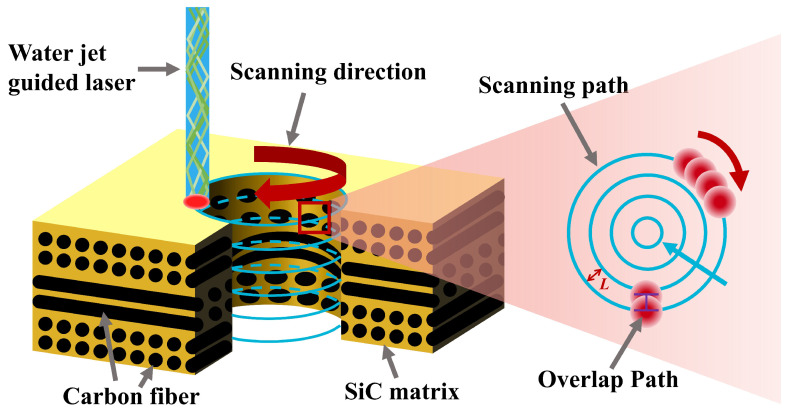
Scanning path of WJGL.

The laser scanning path during drilling was a top-down multilayer concentric circle filling path, as shown in Figure 2. Due to the characteristics of total reflection transmission of the laser in the water jet, the laser focus position does not need to be adjusted during the scanning process [30,31]. The diameter of the processed holes was fixed at 500 µm. The concentric circles were fixed at four. The laser scanned concentric circles from outside to inside and then returned to complete a cycle. When scanning adjacent concentric circles, the overlap area between the water jets is the overlap path, and the ratio of its width to the diameter of the water jet is the path overlap ratio. The path overlap ratio can be adjusted by adjusting the distance *L* between adjacent concentric circles.

After WJGL drilling, the surface morphology and 3D contours of the micro-holes were observed and the micro-hole depths were measured using a laser confocal microscope (Keyence VX-200, Keyence Co., Osaka, Japan) at 500× magnification. The cross-sectional microstructure of the micro-holes was observed and elemental distribution was analyzed with a scanning electron microscope (Regulus-8230, Hitachi, Ltd., Tokyo, Japan). The splashing morphologies were captured with a high-speed camera (Qian Yan Lang X213M, Hefei Zhongke Junda Vision Technology Co., Hefei, China) at the rate of 1000 frames per second with 1280 × 1024 pixels.

## 3. Results and Discussion

Different drilling depths were obtained by varying the level of each factor in the experiment. The significance of the factors on drilling depth was evaluated by the analysis of variance and range of it. From the experimental study, the variance of the seven factors, processing time, power density, water jet speed, scanning speed, path overlap ratio, pulse frequency, and helium pressure on drilling depth were 32,955.6 µm, 23,980.4 µm, 18,294.5 µm, 1274.8 µm, 811.8 µm, 2360.0 µm, and 5849.8 µm, respectively, as shown in Table 3. Their ranges are 536.3 µm, 491.8 µm, 378.4 µm, 97.1 µm, 85.6 µm, 136.9 µm, and 222.8 µm, respectively. These factors can be arranged in descending order of the variance and range, yielding the first three factors with greater effect and the last four factors with less effect. Therefore, the processing time, power density, and water jet speed were identified as significant factors, while the scanning speed, path overlap ratio, pulse frequency, and helium pressure were considered non-significant factors.

### 3.1. Non-Significant Factors on Drilling Depths

#### 3.1.1. Effect of Scanning Speed

**Figure 3:**
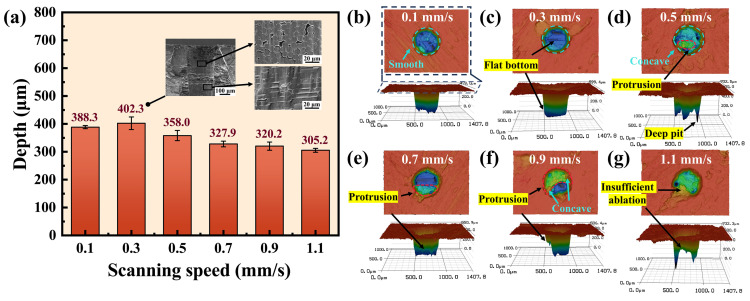
Depth and morphology of micro-holes at different scanning speeds. (**a**) Depth of micro-holes at different scanning speeds. (**b**–**g**) The entrance morphologies and 3D contours of the holes at scanning speeds of 0.1 mm/s, 0.3 mm/s, 0.5 mm/s, 0.7 mm/s, 0.9 mm/s, and 1.1 mm/s, respectively.

As shown in Figure 3a, the micro-hole depth exhibits a primary increase followed by a decrease as the scanning speed increases from 0.1 mm/s to 1.1 mm/s. When the scanning speed was 0.1 mm/s, it took a long time for a single scanning cycle, resulting in low processing efficiency. The maximum processing depth of 402.3 µm was achieved when the scanning speed was 0.3 mm/s. As the scanning speed increased, the number of pulses per unit area and the overlap of adjacent pulses decreased, which reduced the laser energy absorbed by the material [32,33]. As a result, the volume of material reaching the ablation threshold decreased and the depth of micro-holes declined. However, since the processing time was fixed at 45 s, the increase in scanning speed raised the number of scanning cycles. Therefore, there was no significant decrease in the depth of the micro-holes.

Figure 3b–g show the entrance morphology and 3D contours of the holes at scanning speeds of 0.1 mm/s, 0.3 mm/s, 0.5 mm/s, 0.7 mm/s, 0.9 mm/s, and 1.1 mm/s, respectively. From Figure 3d, it is evident that there are a few deep pits with small areas that may exist at the bottom of the hole, which may be attributed to the uneven distribution of pores and carbon fibers within the $C_{f}/\mathrm{SiC}$ composites [34]. Although the maximum depth is shown in the 3D contour, the deep pits were excluded in the analysis of micro-hole depths. From Figure 3b,c, it can be seen that the entrance contours of the ablated holes were smooth and the bottoms were flat when the scanning speed was 0.1 mm/s and 0.3 mm/s. However, as the scanning speed increased, the entrance contour appeared to be concave and a protrusion appeared at the bottom of the hole, and the area and height of the protrusion gradually increased, as shown in Figure 3d–f. At the scanning speed of 1.1 mm/s, there was insufficient ablation in the hole, as shown in Figure 3g. Due to the increased scanning speed, part of the material did not absorb enough laser energy to reach its ablation threshold and therefore remained in the hole.

The cross-sectional micro-morphology in Figure 3a demonstrates the exceptional cleanness of the sidewall processed by the WJGL at the scanning speed of 0.3 mm/s. The cut of transversal and longitudinal carbon fibers was smooth and no thermal damage or debris was observed in the processed area, which was similar to the cold ablation of femtosecond laser [35]. This result indicates that high-quality processing of $C_{f}/\mathrm{SiC}$ composites can be achieved by WJGL.

#### 3.1.2. Effect of Path Overlap Ratio

**Figure 4:**
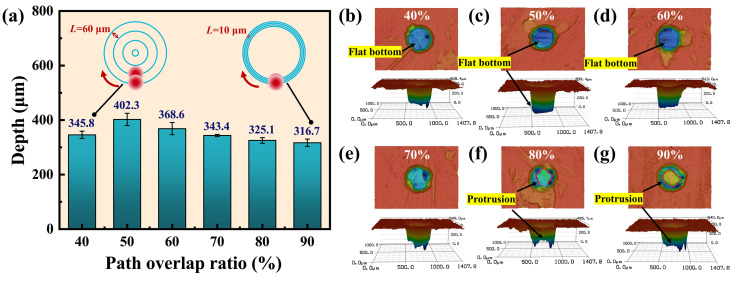
Depth and morphology of micro-holes at different path overlap ratios. (**a**) Depth of micro-holes at different path overlap ratios. (**b**–**g**) The entrance morphologies and 3D contours of the holes at path overlap ratios of 40%, 50%, 60%, 70%, 80%, and 90%, respectively.

Figure 4a shows the effect of the path overlap ratio on the micro-hole depth, which exhibits an initial increase followed by a decrease with the path overlap ratio. When the path overlap ratio was reduced from 50% to 40%, the micro-hole depth decreased from 402.3 µm to 345.8 µm. The reduced overlap between scanning paths led to an extended distance between adjacent concentric circles. As a result, less laser energy was absorbed per unit area of the material within a single cycle, leading to a reduction in the depth of processing. As the path overlap ratio increased from 50% to 90%, the micro-hole depth gradually decreased to 316.7 µm. The reduction in the distance between adjacent concentric circles provided a longer ablation of the material farther from the center of the circle. Consequently, the ablation depth near the edge of the hole was greater than that in the center of it.

Figure 4b–g show the entrance morphologies and 3D contours of the holes at path overlap ratios of 40%, 50%, 60%, 70%, 80%, and 90%, respectively. When the path overlap ratio was lower than 70%, the ablation in the hole was uniform and the bottom of the hole was flat, as depicted in Figure 4b–d, because the hole surface was completely covered by the concentric circle path. And since the diameter of the inner concentric circles was smaller, the number of scanning cycles increased. When the path overlap ratio reached 80% and above, protrusions started to appear in the center of the holes, as shown in Figure 4f,g. At this point, the surface of the holes could not be completely covered by the concentric circles of the path, and the material in the center of the hole was partially removed by heat conduction.

#### 3.1.3. Effect of Pulse Frequency

**Figure 5:**
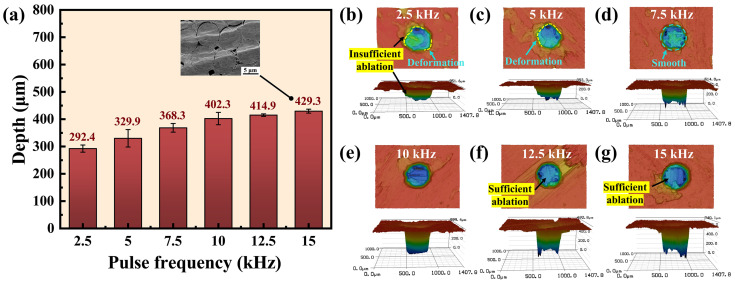
Depth and morphology of micro-holes at different pulse frequencies. (**a**) Depth of micro-holes at different pulse frequencies. (**b**–**g**) The entrance morphologies and 3D contours of the holes at pulse frequencies of 2.5 kHz, 5 kHz, 7.5 kHz, 10 kHz, 12.5 kHz, and 15 kHz, respectively.

As shown in Figure 5a, the depth of the micro-hole increased with pulse frequency. When the pulse frequency was increased from 2.5 kHz to 15 kHz, there was a corresponding increase in hole depth from 292.4 µm to 429.3 µm, reflecting a growth of 31.8%. Since the power density was kept constant, the increased pulse frequency raised the number of pulses per second radiated on the material without reducing the single-pulse energy. Therefore, the laser energy absorbed by the material per second was increased, resulting in an augmented ablation depth. However, the micro-hole depth was not proportionally increased with pulse frequency. At high pulse frequencies, a significant proportion of laser pulses were absorbed and reflected by the insufficiently ablated debris and bubbles produced by material sublimation [36]. In addition, the laser pulses were absorbed by the plasma generated in the processed area [37].

At the pulse frequency of 2.5 kHz, insufficient ablation occurred and the entrance contour exhibited deformation, as shown in Figure 5b. Even when increased to 5 kHz, the entrance remained in deformation, as shown in Figure 5c. The low pulse frequency resulted in a limited amount of material being removed and therefore the entrance was deviated from the circle. However, the entrance and interior of the hole exhibited a smooth and sufficient ablation, as depicted in Figure 5d–g, when the pulse frequency exceeded 5 kHz.

From the microstructure of the carbon fibers cut at the frequency of 15 kHz in Figure 5a, it is evident that there was no molten layer or debris on the fiber surface, indicating a high cutting quality. It is worth noting that the study of Xing et al. [38] showed that a large amount of melt and recast layers were observed in the processed area when the pulse frequency was increased from 5 kHz to 15 kHz while cutting ceramic composites with a nanosecond laser. The heat accumulation on the machined surface increased due to the growing number of pulses deposited per unit area and the shortening of the gap between adjacent pulses, leading to more thermal defects. However, during WJGL processing, the water jet prevented thermal damage by cooling the material between pulses and scouring the molten materials generated by laser ablation [39]. This result demonstrates the superiority of WJGL processing of ceramic composites.

#### 3.1.4. Effect of Helium Pressure

**Figure 6:**
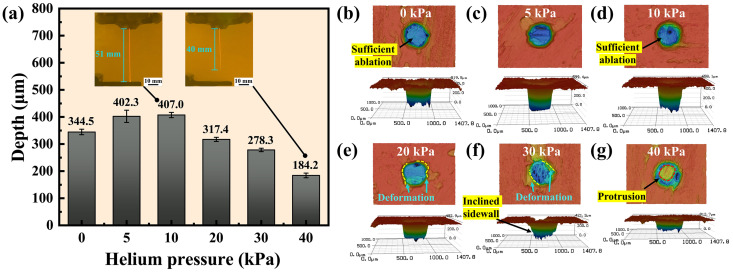
Depth and morphology of micro-holes at different helium pressures. (**a**) Depth of micro-holes at different helium pressures. (**b**–**g**) The entrance morphologies and 3D contours of the holes at helium pressures of 0 kPa, 5 kPa, 10 kPa, 20 kPa, 30 kPa, and 40 kPa, respectively.

In the experiment, helium was used as an auxiliary gas to coaxially surround the water jet, thereby mitigating the interaction between the water jet and ambient air and enhancing the length of laminar flow [40]. The impact of helium pressure on micro-hole depth is illustrated in Figure 6a, with the absence of helium assistance represented by the pressure of 0 kPa. The micro-hole depth was 344.5 µm without helium assistance, while the maximum depth of 407.0 µm was achieved with the helium pressure of 10 kPa, representing an improvement of 18.1%. Since the distance between the nozzle and the workpiece was 25 mm, the water jet maintained a steady laminar flow over this length, resulting in the marginal increase in the processing depth. However, as the helium pressure increased from 10 kPa to 40 kPa, the micro-hole depth decreased from 407.0 µm to 184.2 µm.

To investigate the effect of helium pressure on WJGL processing, the laser transmission length in the water jet at the helium pressures of 10 kPa and 40 kPa was captured with a camera, as shown in Figure 6a. It is obvious that the laser transmission length in the water jet was able to reach 51 mm at a helium pressure of 10 kPa, while the transmission length was only 40 mm at the helium pressure of 40 kPa. The Reynolds number is an important indicator for assessing the stability of the water jet and the auxiliary gas. According to Lasheras et al. [41], when a gas is injected coaxially with the water jet, the Reynolds numbers of the water jet and the auxiliary gas are calculated as follows:

(2) $${Re}_{w}=\frac{\rho_{w}\nu_{w}d_{w}}{\mu_{w}}$$

(3) $$Re_{g}=\frac{\rho_{g}\nu_{g}d_{g}}{\mu_{g}}$$

where $Re_{w}$ and $Re_{g}$ are the Reynolds numbers of the water jet and the gas, respectively. $\rho_{w}$ and $\rho_{g}$, $v_{w}$ and $v_{g}$, $d_{w}$ and $d_{g}$, and $\mu_{w}$ and $\mu_{g}$ are the densities, velocities, equivalent diameters, and dynamic viscosities of the water jet and the gas, respectively.

The velocity of the helium increased with the growth of helium pressure, and the flow transitioned to turbulence when $Re_{g}$ exceeded a critical value. During this period, the helium exhibited erratic movements and interacted with the water jet, resulting in a disturbance on its surface. The disturbance propagated downwards along the surface of the water jet, ultimately resulting in the fragmentation of the water jet. The laser transmission over the surface of the disturbed water jet was affected, resulting in a decrease in the processing depth.

As depicted in Figure 6b–d, the interior of the micro-holes was sufficiently ablated by WJGL when the helium pressure was 0 to 10 kPa, obtaining smooth entrances. However, when the helium pressure was above 10 kPa, the entrance contours underwent deformation, and protrusions and inclined sidewalls occurred due to insufficient ablation, as shown in Figure 6e–g.

High processing efficiency can be achieved without helium assistance during drilling at small depths. Due to the high price of helium, it is possible to process without gas assistance to save costs. Therefore, it is crucial to ascertain the optimal gas pressure for micro-hole processing.

### 3.2. Significant Factors for Drilling Depth

#### 3.2.1. Effect of Processing Time

**Figure 7:**
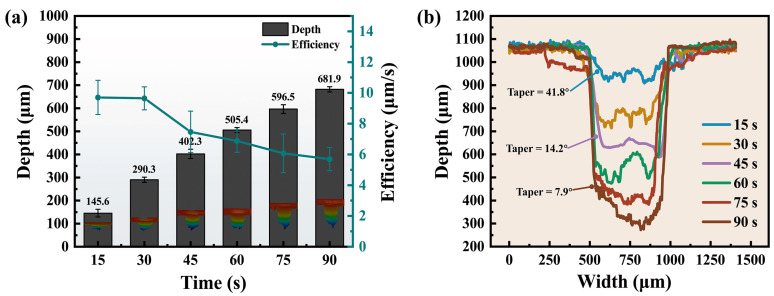
Depth and contour of micro-holes under different processing times. (**a**) Micro-hole depths at different processing times. (**b**) Cross-sectional contours of the micro-holes at different processing times.

Figure 7a shows the relationship between micro-hole depth and processing time. When the processing time increased from 15 to 90 s, the hole depth was significantly increased from 145.6 µm to 681.9 µm. The increase in processing time resulted in a corresponding rise in the number of scanning cycles, leading to enhanced material ablation and consequently an increased depth of ablation. However, the pursuit of minimizing processing time while achieving micro-hole drilling should be emphasized. The efficiency of processing was defined as the drilling depth per second in each increasing 15 s. It can be seen from Figure 7a that the efficiency has been decreasing from 9.7 µm/s at 15 s to 5.7 µm/s at 90 s, indicating a reduction of 41.2%.

The decrease in processing efficiency was attributed to a multitude of factors. The water jet would rebound upwards after reaching the bottom of the hole. The rebound process was characterized by the high flow velocity and the low air pressure surrounding the water jet, which resulted in the convergence of rebound water towards the water jet and subsequently led to fragmentation at the bottom of the water jet [42]. As the processing time grew, the depth of the micro-hole increased and the flow of water through the blind holes became more complicated. The rebound water, upon impacting the sidewall of the hole, may subsequently interact with the water jet, thereby exacerbating its instability. In addition, bubbles may be generated during water jet fragmentation as well as material evaporation under intense laser radiation, inducing cavitation effects. The laser was scattered by the bubbles in the water, which reduced the laser power [36]. As the depth of the hole increased, inadequate drainage at the bottom hindered prompt water discharge, resulting in challenges for the bubbles released from the hole [42].

In addition, it can be seen from the cross-sectional contours of the micro-holes in Figure 7b that the sidewall taper gradually decreased from 41.8° to 7.9° as the processing time increased. A significant amount of time during drilling was spent on reducing the sidewall taper. The sidewall taper resulted from the higher static pressure and laser power density in the central region of the water jet compared to its periphery [43]. WJGL continuously ablated the inclined sidewalls to achieve greater depth. Unlike the circular spot irradiated on a horizontal surface, the irradiation was elliptical on the inclined sidewall. The irradiated area of WJGL gradually increased as the sidewall taper decreased. According to Equation (1), the laser power density was reduced. As a result, more time was consumed for sidewall ablation when dealing with deeper micro-holes, which was one of the factors that contributed to the reduced processing efficiency.

#### 3.2.2. Effect of Power Density

**Figure 8:**
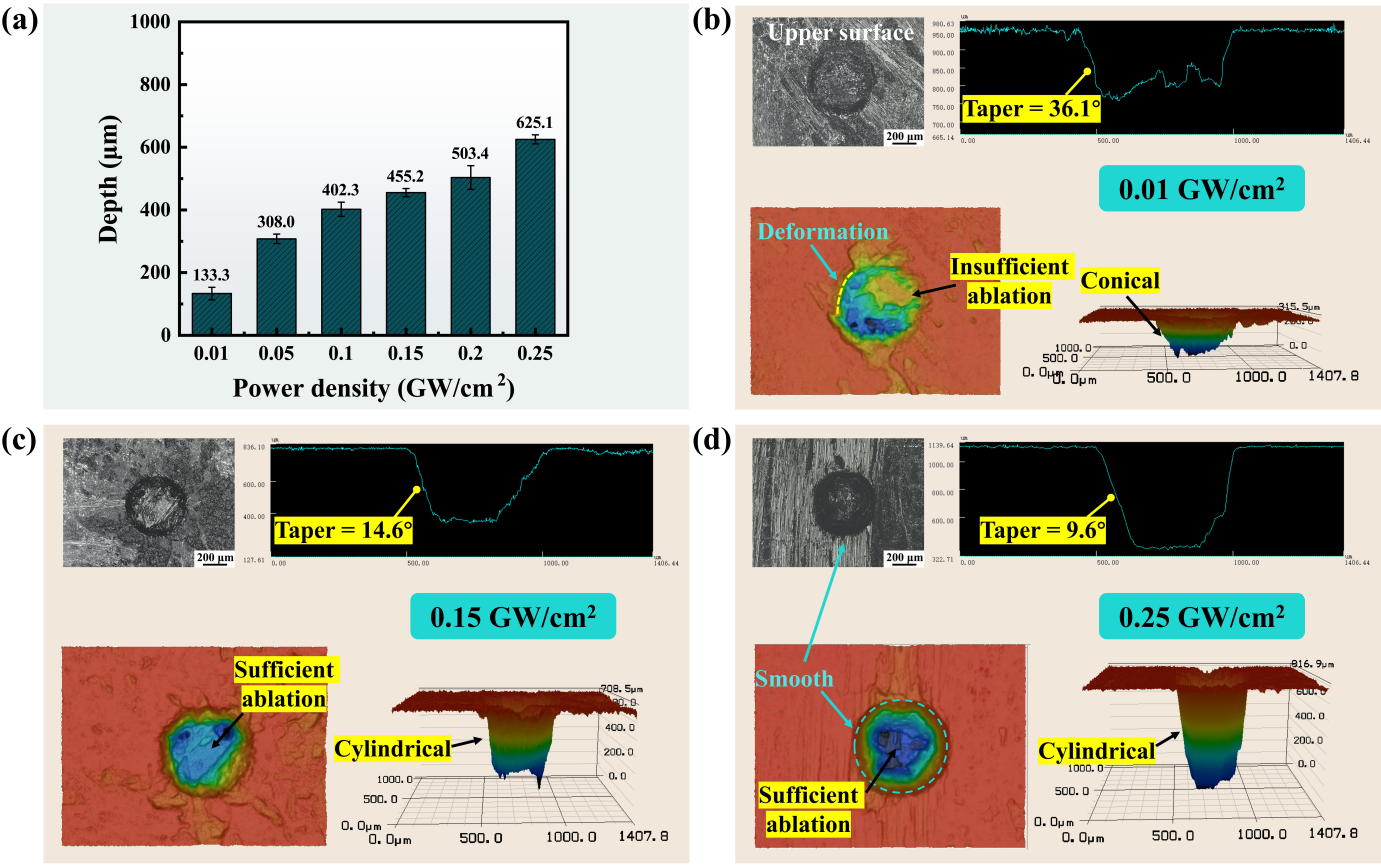
Micro-hole depth and morphology at different power densities. (**a**) Micro-hole depths at different power densities. (**b**–**d**) Hole entrance morphology, cross-sectional contours, and 3D contours at the power densities of 0.01 GW/cm², 0.15 GW/cm², and 0.25 GW/cm², respectively.

The enhancement of laser power density is pivotal for augmenting the capability of deep-hole processing. The material can only be removed when the power density reaches the ablation threshold. As shown in Figure 8a, the micro-hole depth exhibited a significant increase as the power density was elevated. When the laser power density was increased from 0.01 GW/cm² to 0.25 GW/cm², the depth increased from 133.3 µm to 625.1 µm, which was improved by 368.9%. The enhancement in power density arose from an augmentation in pulse energy, thereby increasing the energy absorption of the material. However, the rate of increase in hole depth exhibited a lower magnitude compared to the rate of increase in power density because the plasma shielding effect was found to be significantly enhanced at high pulse energies, leading to a more pronounced attenuation of the laser energy [44].

Figure 8b–d illustrate the upper surface morphologies and 3D contours of the micro-holes at power densities of 0.01 GW/cm², 0.15 GW/cm², and 0.25 GW/cm², respectively. It is evident from Figure 8b that insufficient ablation occurred when the power density was low, resulting in tapered sidewalls and a conical-shaped ablated hole. As shown in Figure 8c,d, as the power density increased, the material within the holes underwent sufficient ablation, and cylindrical holes were formed. Moreover, the sidewall taper was reduced from 36.1° to 9.6°, a reduction of 73.4%. From the upper surface morphologies of the holes, it can be seen that there was no fiber pull-out or breakage at the entrance of the holes.

**Figure 9:**
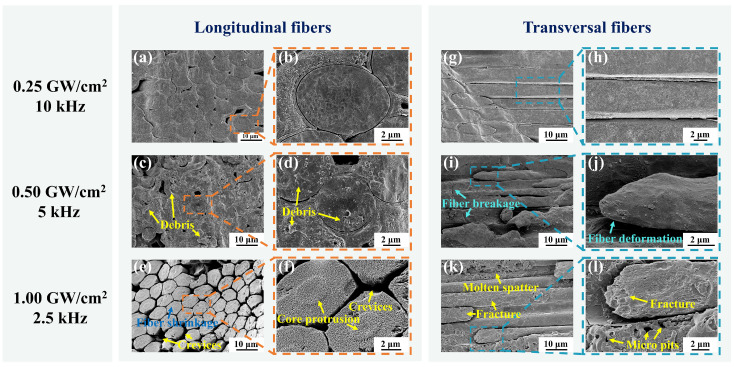
Microstructure of cross-sectional carbon fibers at different power densities. (**a**,**b**) Microstructure of longitudinal carbon fibers at the power density of 0.25 GW/cm². (**c**,**d**) Microstructure of longitudinal carbon fibers at the power density of 0.50 GW/cm². (**e**,**f**) Microstructure of longitudinal carbon fibers at the power density of 1.00 GW/cm². (**g**,**h**) Microstructure of transversal carbon fibers at the power density of 0.25 GW/cm². (**i**,**j**) Microstructure of transversal carbon fibers at the power density of 0.50 GW/cm². (**k**,**l**) Microstructure of transversal carbon fibers at the power density of 1.00 GW/cm².

As demonstrated in Figure 9, the microstructure of cross-sectional carbon fibers at different power densities was investigated. Due to the limited power of the laser source employed in the experiment, the pulse frequency was reduced to achieve power densities of 0.50 GW/cm² and 1.00 GW/cm² while maintaining the fixed laser power of 15 W. As shown in Figure 9a,b, a neat and clean cut of the longitudinal carbon fibers was obtained at the power density of 0.25 GW/cm². However, when the power density was increased to 0.50 GW/cm², a large amount of debris appeared on the fiber surface, as illustrated in Figure 9c,d. Furthermore, the results depicted in Figure 9e,f demonstrate that the carbon fiber experienced shrinkage and core protrusion upon increasing the power density to 1.00 GW/cm². Moreover, crevices were observed between the fibers. The heat was not able to be fully dissipated by the water jet at high power densities, thereby resulting in thermal damage. Fiber shrinkage and crevices were attributed to the higher sublimation temperature of the carbon fibers than the silicon carbide and pyrolytic carbon interface layers [45]. The energy at the edges of the water jet sublimated the silicon carbide matrix and the pyrolytic carbon interface layer but not the carbon fibers. In addition, the decomposition temperature of the core is higher than that of the outermost layer in the carbon fiber. As a result, the carbon fiber shrank, and the core protruded [46].

As illustrated in Figure 9g,h, the transversal carbon fibers cut at the power density of 0.25 GW/cm² also exhibited excellent smoothness and cleanness. In contrast, fiber deformation and breakage were observed at the power density of 0.50 GW/cm², as shown in Figure 9i,j. When the laser energy increased, since the axial thermal conductivity of carbon fiber was higher than the radial direction [47], the heat was prone to propagate along the axial direction of the fiber and generated thermal stresses, leading to fiber deformation and breakage. As indicated in Figure 9k,l, molten spatter, fiber fracture, and micro pits were observed on the cut at the power density of 1.00 GW/cm². During processing at high power densities, the sharp absorption and explosion of laser energy by the plasma and the shock pressure generated by the rupture of microbubbles may lead to micro pits and fiber fracture [48]. Moreover, the heightened power density led to a propensity for laser energy deposition on the nozzle edge, thereby increasing the possibility of nozzle damage and subsequent additional costs.

In conclusion, the increase in power density led to a greater micro-hole depth but concurrently resulted in more thermal defects. Therefore, the selection of an appropriate power density is crucial in attaining efficient and minimally damaging processing.

#### 3.2.3. Effect of Water Jet Speed

**Figure 10:**
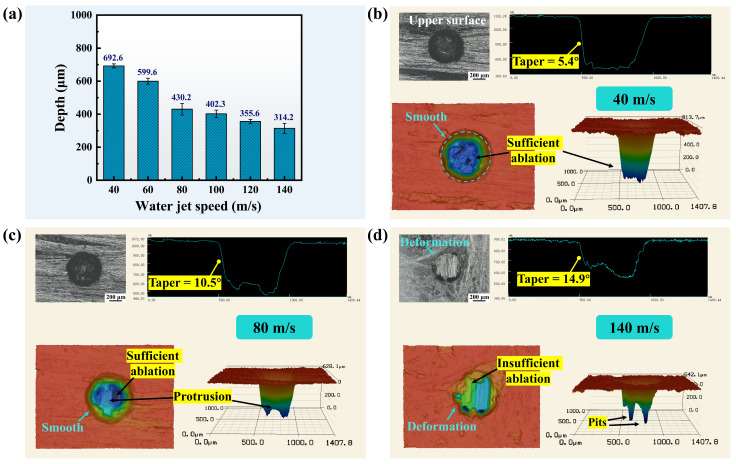
Depth and morphology of micro-holes at different water jet speeds. (**a**) Micro-hole depths at different water jet speeds. (**b**–**d**) Hole entrance morphology, cross-sectional contours, and 3D contours at the water jet speeds of 40 m/s, 80 m/s, and 140 m/s, respectively.

The effect of water jet speed on the depth of micro-holes is shown in Figure 10a. It is evident that the micro-hole depth decreased significantly as the water jet speed increased. When the water jet speed was increased from 40 m/s to 140 m/s, the micro-hole depth decreased from 692.6 µm to 314.2 µm, a reduction of 54.6%. In addition, the hole entrance and 3D contour were also deformed. As shown in Figure 10b, when the water jet speed was 40 m/s, the entrance was smooth and the material ablation was sufficient. As the water jet speed increased to 80 m/s, a protrusion appeared in the hole, as shown in Figure 10c. At the water jet speed of 140 m/s, the entrance of the hole exhibited deformation. Meanwhile, insufficient ablation and deep pits occurred within the hole, as shown in Figure 10d. Moreover, the sidewall taper increased from 5.4° to 14.9°.

**Figure 11:**
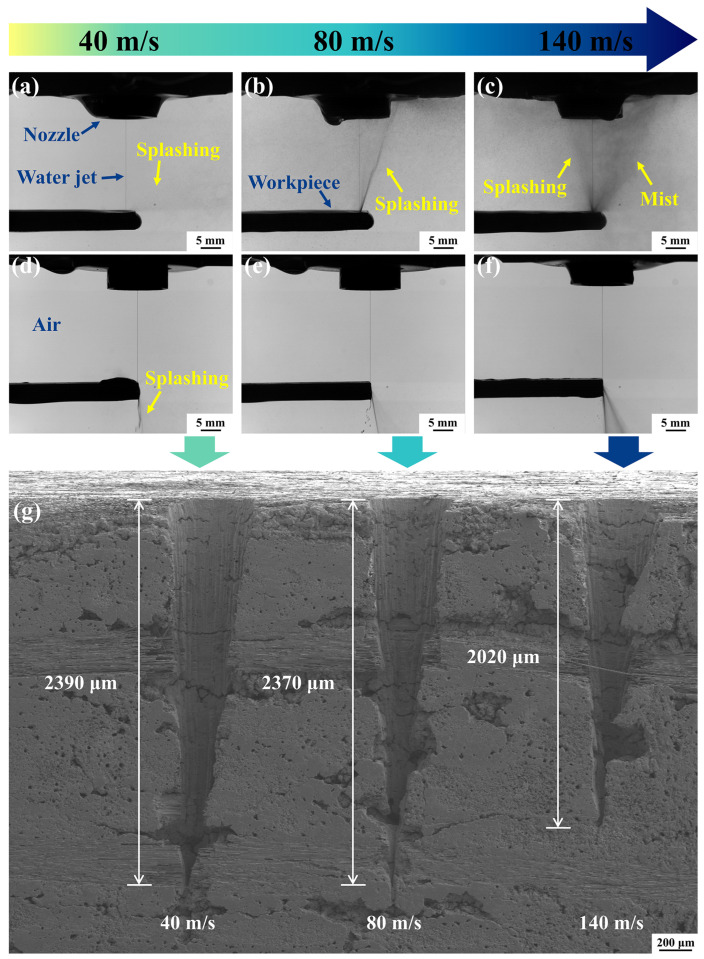
Splashing morphologies for drilling inside and on the edge of the workpiece. (**a**–**c**) Splashing morphologies at the water jet speeds of 40 m/s, 80 m/s, and 140 m/s for drilling inside the workpiece, respectively. (**d**–**f**) Splashing morphologies at the water jet speeds of 40 m/s, 80 m/s, and 140 m/s for drilling on the edge of the workpiece, respectively. (**g**) Micro-hole depths at the water jet speeds of 40 m/s, 80 m/s, and 140 m/s for drilling on the edge of the workpiece.

The reduction in micro-hole depth may be attributed to the phenomenon of water splashing occurring during the processing. During micro-hole drilling, splashing was formed by the water jet impinging on the bottom of the hole and subsequently ejecting along the sidewalls into the air. As illustrated in Figure 11a–c, as the water jet speed increased from 40 m/s to 140 m/s, the water jet impinged on the bottom of the micro-hole at a greater speed and sprayed more splashing droplets upwards, creating a larger mist in the air. The impingement of numerous splashing droplets on the water jet may result in deformation or even breakage of the water jet, which significantly affected the laser transmission [49]. In addition, the water jet was susceptible to being impacted and broken up by the water bouncing off the bottom of the hole with increased speed. To validate the role of splashing in the reduction in micro-hole depth, experiments were conducted by drilling on the edge of the workpiece. The center of the path was fixed on the edge of the workpiece. Figure 11d–f show that splashing was ejected downward while drilling on the edge at the water jet speeds of 40 m/s, 80 m/s, and 140 m/s, respectively. Therefore, there was no splashing and mist in the air. As illustrated in Figure 11g, the depths of drilling on the edge were 2390 µm, 2370 µm, and 2020 µm at the water jet speeds of 40 m/s, 80 m/s, and 140 m/s, respectively. The micro-hole depth decreased by only 15.4% when the water jet speed was increased from 40 m/s to 140 m/s. Therefore, the splashing resulting from the increasing water jet speed was identified as a significant factor influencing the drilling depth.

**Figure 12:**
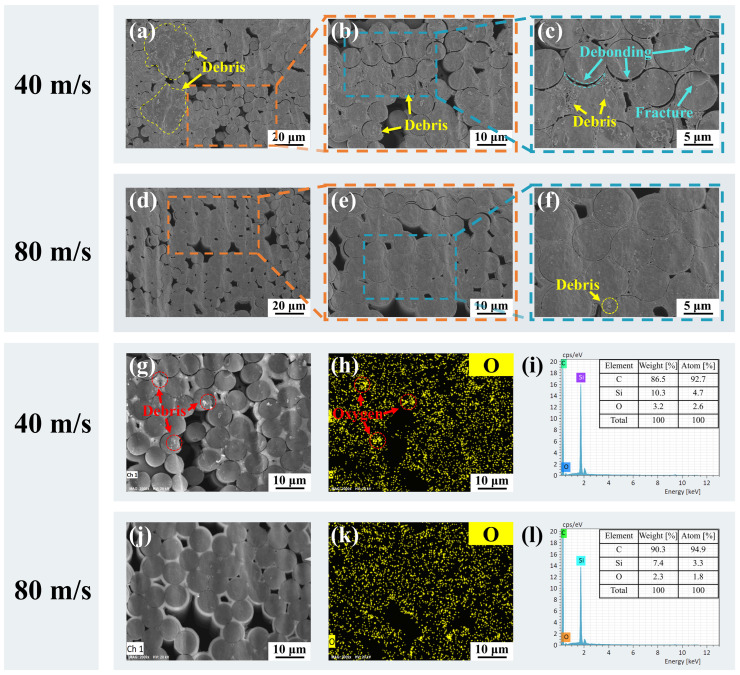
Cross-sectional microstructure analysis for different water jet speeds. (**a**–**c**) Cross-sectional microstructure at the water jet speed of 40 m/s. (**d**–**f**) Cross-sectional microstructure at the water jet speed of 80 m/s. (**g**–**i**) EDS spectra analysis of the cross-section at the water jet speed of 40 m/s. (**j**–**l**) EDS spectra analysis of the cross-section at the water jet speed of 80 m/s.

It is worth noting that the decrease in water jet speed could affect the drilling quality. As depicted in Figure 12a, debris with a diameter of about 20 µm was observed in the processed cross-section at the water jet speed of 40 m/s. As can be seen from the enlarged microstructural view in Figure 12b,c, a large amount of debris with small diameters was present on the surface of the fibers, accompanied by defects of fiber fracture and debonding. However, when the water jet speed was increased to 80 m/s, the surface of the cut appeared exceptionally clean, and only debris with a diameter of less than 1 µm was observed, as shown in Figure 12d–f. The element distribution was analyzed for cutting surfaces with water jet speeds of 40 m/s and 80 m/s, as shown in Figure 12g–i and j–l, respectively. The comparison of Figure 12i,l reveals that the concentration of oxygen and silicon elements was higher at the water jet speed of 40 m/s compared to the water jet speed of 80 m/s. The phenomenon of oxygen element aggregation can be observed in Figure 12h, the position of which corresponds to the position of the debris in Figure 12g. Therefore, the debris may be the oxide of silicon. The scouring and convective cooling effects of the water jet were enhanced when the water jet speed was increased to 80 m/s, preventing heat accumulation and debris adherence on the cut [50].

In conclusion, the decrease in the water jet speed and the increase in the power density contributed to greater processing efficiency but may lead to the additional thermal damage on the cut. According to the experimental results presented in Section 3.2.2 and Section 3.2.3, the higher processing efficiency and lower thermal damage could be achieved simultaneously when the power density was 0.25 GW/cm² and the water jet speed was 80 m/s. Therefore, these two processing parameters were selected for deep-hole drilling in the experiment.

### 3.3. Micro Deep-Hole Drilling

**Figure 13:**
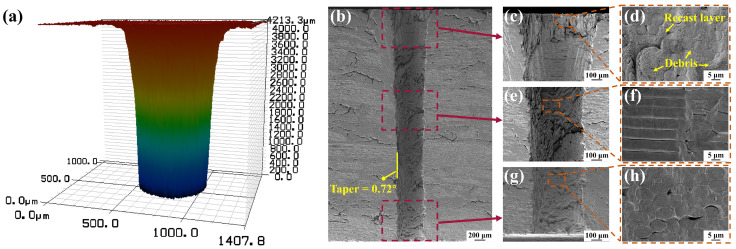
Morphology of the micro-hole with a depth of 4.1 mm drilled by WJGL. (**a**) The 3D contour of the micro-hole. (**b**) Cross-sectional morphology of the micro-hole. (**c**) Cross-sectional morphology of the entrance. (**d**) Microstructure of the entrance. (**e**) Cross-sectional morphology of the middle region. (**f**) Microstructure of the middle region. (**g**) Cross-sectional morphology of the exit. (**h**) Microstructure of the exit.

The micro deep-hole drilling was conducted by optimizing the influential factors. The power density utilized was 0.25 GW/cm², accompanied by the pulse frequency of 10 kHz, the water jet speed of 80 m/s, the scanning speed of 0.3 mm/s, the path overlap ratio of 50%, and the helium pressure set at 10 kPa. The micro-hole with an average diameter of 510 µm, a depth of 4.1 mm, and a depth-to-diameter ratio of 8.03 was obtained, as illustrated in Figure 13a.

As shown in Figure 13b, the cross-section of the micro-hole exhibited no discernible heat-affected zone and featured a sidewall taper of only 0.72°. The diameter of the hole at the entrance was larger than at the internal and exit, which could be attributed to the longer processing time at the entrance, resulting in an extended impact of the water jet and heat transfer. In Figure 13d, it can be seen that the recast layers and debris existed on the fiber surface at the entrance, but the fibers were not deformed. The possible reasons for this phenomenon may be that the melt inside the hole was entrained by the water jet and deposited near the entrance during processing and that the longer processing time resulted in a minor amount of heat accumulation. In addition, the middle and exit region of the cross-section demonstrated exceptional processing quality, characterized by a smooth and clean cut without thermal defects, as shown in Figure 13f,h. In conclusion, high-quality micro-hole drilling of $C_{f}/\mathrm{SiC}$ composites could be achieved by WJGL with the selection of appropriate parameters.

## 4. Conclusions

In this study, the effects of seven factors, namely processing time, power density, water jet speed, scanning speed, path overlap ratio, pulse frequency, and helium pressure, on the micro-hole depth and morphology of $C_{f}/\mathrm{SiC}$ composites drilled by WJGL were investigated. These factors can be classified into significant and non-significant categories. The significant factors include processing time, power density, and water jet speed. Their effects on the micro-hole processing can be summarized as follows:

- The processing efficiency declined with the increasing processing time. When the processing time was increased from 15 to 90 s, the processing efficiency decreased from 9.7 µm/s to 5.7 µm/s, a decrease of 41.2%;
- The increase in power density is essential for deep-hole drilling. As the power density increased from 0.01 GW/cm² to 0.25 GW/cm², the micro-hole depth increased from 133.3 µm to 625.1 µm. However, when the power density exceeded 0.25 GW/cm², thermal defects appeared on the cut because the residual heat could not be fully absorbed by the water jet;
- The increase in water jet speed facilitated the improvement of processing quality while resulting in a decrease in drilling depth. As the water jet speed increased from 40 m/s to 140 m/s, the splashing became progressively severe and interfered with the water jet, resulting in a decrease of 54.6% in the micro-hole depth. However, the elevated water jet speeds contribute to the cooling of the processing area and reduction in oxide accumulation.

The non-significant factors include scanning speed, path overlap ratio, pulse frequency, and helium pressure. The effects of these factors on micro-hole processing can be summarized as follows:

- The depth of the micro-holes exhibited a slight increase followed by a subsequent decrease as the scanning speed, path overlap ratio, and helium pressure were increased. The optimal scanning speed of 0.3 mm/s, a path overlap ratio of 50%, and a helium pressure of 10 kPa were determined to achieve the maximum drilling depth. In terms of drilling quality, as the scanning speed, path overlap ratio, and helium pressure continued to increase, the entrance of the hole exhibited deformation while insufficient ablation and protrusion formed within it;
- By increasing the pulse frequency, the drilling depth was increased, achieving a smoother entrance and sufficient ablation. However, when the pulse frequency was increased from 2.5 kHz to 15 kHz, the hole depth was only improved by 31.8%.

Based on the optimization of the processing parameters, the micro deep hole with an average diameter of 510 µm, a depth of 4.1 mm, a depth-to-diameter ratio of 8.03, and a sidewall taper of merely 0.72° was achieved. The microstructural observations of the cross-section revealed that the quality exhibited higher levels in the middle region and at the exit compared to those observed at the entrance. No thermal defects were observed in the middle region of the cross-section or at the exit, and the cut was smooth and clean. Therefore, the WJGL processing technology enables the realization of high quality micro deep-hole processing for $C_{f}/\mathrm{SiC}$ composites.

Due to the limitations of the laser and water pump pressure, higher laser power densities and water jet speeds could not be obtained in this study. Therefore, subsequent research can be carried out with higher levels of the parameters and the investigation of their potential interactions.

## Figures and Tables

**Table 1:** Characteristic parameters of the $C_{f}/\mathrm{SiC}$ composites at room temperature.

Properties	Parameters	Units
Diameter of carbon fiber	6–8	µm
Thickness of PyC	∼0.3	µm
Density	1.7	$g/{\mathrm{cm}}^{3}$
Fiber volume fraction	∼40	%
Porosity	∼18	%
Tensile strength	>120	MPa
Bending strength	>250	MPa
Interlayer thermal conductivity	∼5	W/(m·K)
Size	58.2 × 10.0 × 4.1	mm

**Table 2:** Experimental parameters.

Parameter	Value	Units
Laser wavelength	532	nm
Pulse width	70–100	ns
Maximum average power	15	W
Repetition frequency	2.5, 5, 7.5, 10, 12.5, 15	kHz
Power density	0.01, 0.05, 0.10, 0.15, 0.20, 0.25	$\mathrm{GW}/{\mathrm{cm}}^{2}$
Water jet speed	40, 60, 80, 100, 120, 140	m/s
Scanning speed	0.1, 0.3, 0.5, 0.7, 0.9, 1.1	mm/s
Path overlap ratio	40, 50, 60, 70, 80, 90	%
Helium pressure	0, 5, 10, 20, 30, 40	kPa
Drilling time	15, 30, 45, 60, 75, 90	s
Nozzle diameter	100	µm

**Table 3:** The variance of each factor on the processing depth.

Factor	Depth 1	Depth 2	Depth 3	Depth 4	Depth 5	Depth 6	Range	Variance
Processing time	145.6	290.3	402.3	505.4	596.5	681.9	536.3	32,955.6
Power density	133.3	308.0	402.3	455.2	503.4	625.1	491.8	23,980.4
Water jet speed	692.6	599.6	430.2	402.3	355.6	314.2	378.4	18,294.5
Scanning speed	388.3	402.3	358.0	327.9	320.2	305.2	97.1	1274.8
Path overlap ratio	345.8	402.3	368.6	343.4	325.1	316.7	85.6	811.8
Pulse frequency	292.4	329.9	368.3	402.3	414.9	429.3	136.9	2360.0
Helium pressure	344.5	402.3	407.0	317.4	278.3	184.2	222.8	5849.8

## Data Availability

The data are contained within the article.

## Abbreviations

The following abbreviations are used in this manuscript:

WJGL	Water jet-guided laser
CFRP	Carbon fiber-reinforced plastics
3D	3-dimensional
CCD	Charge-coupled device
